# 

**Published:** 1832

**Authors:** 


### CONTENTS OF VOL. VI.

### NO. I.

### ESSAYS AND CASES.

I.—A Sketch of the Climate of the Valley of the Mississippi. By Daniel Drake, M. D. - - 9
II.—Facts and Cases, reported for the Western Journal. By William Maclay Awl, of Somerset, Ohio. - 23
III.—Inquiries into the Origin and the mode of Expulsion of the Ascaris Lumbricoides. By George R. Morton, M. D., Coshocton, Ohio. - - - 28
IV.—An Essay on Erysipelas, read before the Cincinnati Medical Society, 1832. By Edwin A. AtLee, M. D. 35
V.—Notices of the Influenza and Meazles, as they appeared at Cincinnati, in 1831 and 1832. By Daniel Drake, M D. - - - - 45

### REVIEWS AND BIBLIOGRAPHICAL NOTICES.

VI.—An account of some of the most important Diseases peculiar to Women. By Robert Gooch, M. D. From the second London edition. Philadelphia: E. L. Carey and A. Hart, Chestnut street—1832. - 53
VII.—Epidemic Cholera—its History and Ætiology. An Eclectic Review. - - - - 78

### MISCELLANEOUS INTELLIGENCE.

I. ANALECTIC.

1. Uterine Irritation.—The Morbid Conditions of the General System and of Particular Organs which result from the continuance of Uterine Irritation. - - - 121
2. M. Delpech on Varicocele. - - - 135
3. On Death from Lithotomy. - - - 137
4. Poisoning from Cantharides. - - - 140
5. Congenital Malformation of the Cornea and Sclerotica. - 140
6. Treatment of Croup. - - - - 141
7. Vesico-Vaginal Fistula successfully treated by the Suture. 141
8. Mr. Duffin's New Shield Pessary. - - - 142
9. Congenital Blindness cured at an advanced age. - 143
10. Œsophagitis Hydrophobia. - - - 144
11. Case of Extra-uterine Fœtation. - - - 145
12. On Death from Syncope.—Observations on Venesection. - 148
13. Heurteloup on Lithotrity.—Construction of Instruments. 149
14. Remarks on Lactation; with Observations on the Healthy and Diseased Conditions of the Breast-milk, &c. &c. - 154

15. Re-union of Divided Parts. - - - 156
16. Deviation of the Menses. - - - - 157
17. Anomalous Menstruation. - - - - 158
18. Constituent parts of Cinchona. - - - 159
19. Lithotrity. - - - - - 159
20. Rare Luxation of the Patella. - - - 159
21. Ossification of the Pericardium. - - - 160
22. Chrystallization of Perchloric Acid. - - 160
23. Hydrocyanic Acid. - - - - 160
24. Cyanide of Potassium. - - - - 160
25. Perchloric Acid as a test for the Mineral Alkalies. - 161

II. ANALYTICAL.

26. Extirpation of the Parotid Gland. - - - 161
27. Dislocation of the Astragalus and subsequent Extraction of the bone—Foot preserved. - - - 163
28. Tracheotomy. - - - - - 164
29. Hypertrophy of the Tongue. - - - 165
30. Wound of the Abdomen and Section of the Intestinal Canal successfully treated on the plan of Ramdohr. - - 166
31. Spontaneous Evolution of the Fœtus. - - 167

---

### NO. II.

### ESSAYS AND CASES.

I.—An inquiry into the Anatomical Condition of the Alimentary Canal in its healthy state. Extracted from the Pathological Anatomy of Professor Andral. - 169
II.—On the Use of Digitalis in Amenorrhea, with cases of of its successful administration. By W. T. S. Cornett, M. D. - - - - 184
III.—Observations on the Modus Operandi of Calomel. By a Correspondent. - - - - 189
IV.—Case of Complete Division of the Tendo Achillis, by the blow of an Axe. Communicated by John L. Richmond, M. D., of Cincinnati. - - 191
VI.—Notices of the Epidemic Constitution of the Summer of 1832, at Cincinnati. By Daniel Drake, M. D. 198

### REVIEWS AND BIBLIOGRAPHICAL NOTICES.

VII.—A Practical Compendium of Midwifery, being the course of Lectures on Midwifery and on the Diseases of Women and Infants, delivered at St. Bartholomew's Hospital. By the late Robert Gooch, M. D. Philadelphia: Carey and Hart—pp. 120, 8vo.—1832. 211
VIII.—A Treatise on the Venereal Diseases of the Eye. By W. M. Lawrence, F. R. S., late professor of Surgery to the Royal College of Surgeons in London, Surgeon to St. Bartholomew's Hospital, &c. - 229

IX.—Surgical Memoirs of the Campaigns of Russia, Germany and France. By Baron D. J. Larrey. - 244
X.—Epidemic Cholera. - - - - 266

### MISCELLANEOUS INTELLIGENCE.

ANALECTIC.

1. Wound of the Head.—Ball extracted from the Brain. - 272
2. Wound of the Head.—Severe Injury of the Head, with loss of Speech and other Nervous Affections. - - 274
3. Wound of the Thorax.—Penetrating Sabre Thrusts. - 277
4. Wound produced by the discharge of a Pistol within the cavity of the Mouth. - - - - 278
5. Thoughts on Insanity. - - - - 280
6. Treatment of Gout. - - - - 282
7. Case of Iliac Aneurism with ligature of the External Iliac Artery. - - - - - 285
8. Re-union of Divided Parts. - - - 288
9. Irreducible Hernia. - - - - 289
10. Symptoms of Aortic Aneurism. - - - 290
11. Partial Fracture of the Long Bones in Children, - 293
12. Rupture of the Kidney. - - - - 295
13. Cholera at Paris. - - - - 298
14. Remarks on the Pathology and Treatment of the Cholera. 303
15. Accidents from the admission of Air into the Veins. - 310
16. Letter on Cholera from Professor Chapman. - - 315
17. Algid Fever of Smyrna compared with the Cholera. - 319
18. Dysentery accompanied with an unusual affection of the Skin. 319
19. Mitchell's Elements of Chemical Science. - - 320

### NO. III.

### ESSAYS AND CASES.

I.—Epidemic Cholera in Cincinnati. By Daniel Drake, M. D. - - - - - - - - - - - 321
II.—Extraordinary Case of Protracted Vomiting, in which Life was prolonged for an unusual length of time without Food. By Daniel Sexton, M. D., of New Harmony, Indiana. - - - - - - - 365
III.—Case of Cholera combined with Symptoms of Remittent Fever. By James C. Finley, M. D. - - - 367
IV.—Notices of Scarlet Fever in Montgomery county. By George Branch, M. D. - - - - - - - 370

### REVIEWS AND BIBLIOGRAPHICAL NOTICES.

V.—Cooper on Dislocations and Fractures of the Joints, 375
VI.—Elements of Chemical Philosophy, on the Basis of Reid, with the requisite Illustrations and Diagrams. By Thomas D. Mitchell, M. D. - - - - - 394
VII.—On Baths and Mineral Waters. In two parts. By John Bell, M. D. - - - - - - - - 409

### MISCELLANEOUS INTELLIGENCE.

### ANALECTIC.

1. Process of preparing pure Muriate of Morphia. - - 425
2. Poisoning by Colchicum. - - - - - 429
3. Experiments with Morphine, - - - - - 435
4. Test for Mercury and its Salts. - - - - 442
5. Facts relating to Hydrophobia. - - - - 443
6. Aneurism from wound of the Brachial Artery. - - 451
7. Chronic Hydrocephalus cured by Puncturing. - - 453
8. Preternatural Lactation. - - - - - - 454
9. Efforts of Nature to establish a New Joint after Dislocation. 457
10. On the Formation of the Capsular Ligaments. - - 459
11. Singular Case of Lacerated Wound. - - - 460
12. On the Sleep of Plants. - - - - - - 462
13. Ilicine—a Remedy in Intermittent Fever. - - 462
14. Regeneration of Nerves. - - - - - 463
15. The Diving Bell a Remedy for certain Diseases, - - 465
16. Observations on Club Feet. - - - - 467
17. The Resurrectionists. - - - - - 469
18. Effects of Darkness in producing Deformity. - - 470
19. Insomnolence cured by Sulphate of Quina. - - 470
20. Treatment of Scrofula by Iodine. - - - - 470
21. Natural History of Man, - - - - - 471
22. Medical Jurisprudence. - - - - - 472
23. Rhinoplastic Operation. - - - - - 472
24. Sex of a Child not discovered until it was 18 years old. - 473
25. Probable Case of Extra-uterine Pregnancy, - - 474
26. Torsion of the Arteries after Surgical Operation. - 474
27. Permanent Contraction of the Fingers cured by dividing the Palmar Aponeurosis. - - - - 475
28. Capsicum in Hemorrhage. - - - - 475
29. The Ornithoryncus Paradoxus. - - - - 476
30. Curious Anomaly in the Distribution of the Blood-vessels. 476
31. Dissection of a person who died of Inanition. - - 477
32. Tumor—The Remains of an old Hernial Sac in the Sheath of the Spermatic Cord. - - - - - - 477
33. Retention of Urine caused by a foreign Body in the Rectum. 478
34. Arresting Hemorrhage. - - - - - 478
35. To protect Iron and Steel from Rust, - - 478
36. Obituary Notice. - - - - - 479

---

### NO. IV.

### ESSAYS AND CASES.

I.—Observations on the Laws of Sympathetic Action in the Animal Œconomy. By James C. Finley, M. D. 481
II.—Observations on Scarlet Fever, as it appeared in Steubenville, Ohio, during the winter of 1831 and 1832. By A. Judkins, M. D. - - - - 494
III.—Report of a Case of a long standing Ulcer, from which were extracted three Teeth, resembling those of the Human Jaw. By James C. Finley, M. D. - - 519

### REVIEWS AND BIBLIOGRAPHICAL NOTICES.

IV.—A Treatise on Fever. By Southwood Smith, M. D., Physician to the London Fever Hospital. Philadelphia: Carey & Lea—pp. 450, 8vo.
Clinical Illustrations of Fever, comprising a Report of the Cases treated at the London Fever Hospital, in 1828 and 1829. By Alexander Tweedie, M. D., Physician to the London Fever Hospital, &c. &c. Philadelphia: Carey & Lea—pp. 150, 8vo. - - - 524
V.—Pathological and Practical Researches on Diseases of the Brain and Spinal Cord. By John Abercrombie, M. D., Fellow of the Royal College of Physicians of Edinburgh, &c., and first Physician to his Majesty in Scotland. Philadelphia: Carey & Lea—pp. 450, 8vo. 548
VI.—A. Cornelii Celsi de Medicina Libri octo; ex recensione Leonardi Targæ.
The Eight Books of Aulus Cornelius Celsus on Medicine; from the Text of Leonardus Targa. - - 574
VII.—A Manual of Surgery, founded upon the principles and practice lately taught by Sir Astley Cooper and Joseph Henry Green, Esq., F. R. S., Professor of Anatomy to the Royal Academy, Surgeon to, and Lecturer on Surgery, St. Thomas's Hospital. Edited by Thomas Castle, F. L. S. Boston: Munroe & Francis. New York: Charles L. Francis. 1833.—pp. 460, 12mo. - - - - - - - - 586

### MISCELLANEOUS INTELLIGENCE.

### I. ANALECTIC.

1. On Nervous Ganglions, and on the Origin and Nature of the Intercostal Nerve. - - - - - 593
2. Case of an Eruptive Disease arising from the use of Cubebs. 595
3. Treatment of Gastrodynia by Dr. Graves. - - 596
4. On the Treatment of Habitual Constipation. - - 597
5. M. Dupuytren on the Consolidation of Fractures of the Neck of the Femur within the Capsule. - - - 600
6. Furious Delirium, consequent on the Repercussion of Erysipelas, cured by recalling the Inflammation. - - 602
7. Lunar Caustic Blister. - - - - - 603
8. New method of operating in Ectropion. - 603
9. Operation of Lithotomy for the Extraction of a Portion of Catheter from the Bladder. - - - - 604
10. Native Lithotomists in India. - - - - 605
11. Cases of Phlebitis—Advantages of Free Openings. - 606
12. Treatment of Croup. - - - - - 609
13. Croup. - - - - - - - 612
14. Affections of the Head produced by Quinine. - - 613
15. Spinal Apoplexy, or Extravasation of Blood in the Spinal Canal. - - - - - - - - 614
16. Destruction of a Portion of the Spinal Cord. - - 615
17. Case of Aphonia, treated by Nitras Argenti. - - 616
18. Case of Rheumatism, treated by the Local Use of the Acetate of Morphia. - - - - - - 617

19. Case of severe Sciatica cured by Blood-letting. - - 617
20. Paralysis of the Portio Dura cured by Moxas. - - 618
21. Vitreous Humor Regenerated. - - - - 618
22. Cases of Vesico-Vaginal and Recto-Vaginal Fistula cured. 619
23. Tic Douloureux of a Stump said to be cured by the removal of a portion of the Median Nerve. - - - 621
24. Diffused Hydrocele of the Cord. - - - - 623
25. Scrofulous Disease of the Bones. - - - - 624
26. Puerperal Peritonitis, followed by Ascites and Spontaneous Perforation of the Abdominal Parietes. - - - 626
27. On large doses of Quinine in Atmospheric Fevers. - 627
28. Obituary Notice of Dr. Spurzheim, from the Funeral Oration of Professor Follen. - - - - - 628

II. ANALYTICAL.

29. Documents relative to the Medical College of Ohio; and the Law regulating the Practice of Physic in Ohio. - - 632

